# A single session of transcranial static magnetic stimulation placed over the visual cortex was not associated with measurable changes in dynamic stability or static postural control in individuals with recurrent lateral ankle sprains: a randomized double-blind placebo-controlled trial

**DOI:** 10.3389/fspor.2026.1918375

**Published:** 2026-10-23

**Authors:** Shunya Nonoyama, Yuka Shimozawa, Tatsuya Mima, Tadao Isaka, Masafumi Terada

**Affiliations:** 1 Faculty of Sport and Health Science, Ritsumeikan University, Kusatsu, Shiga, Japan; 2 Research Organization of Science and Technology, Ritsumeikan University, Kusatsu, Shiga, Japan; 3 Institute of Advanced Research for Sport and Health Science, Ritsumeikan University, Kusatsu, Shiga, Japan; 4 The Graduate School of Core Ethics and Frontier Sciences, Ritsumeikan University, Kyoto, Kyoto, Japan

**Keywords:** ankle injury, non-invasive brain stimulation, postural control, sensorimotor function, sensory reweighting

## Abstract

**Introduction:**

Diminished postural control is a motor-behavioral impairment associated with recurrent lateral ankle sprains. Individuals with recurrent lateral ankle sprains rely on visual cues and exhibit cortical overactivation in the visual cortex, which may be linked to poor postural control. Transcranial static magnetic stimulation (tSMS), which reduces visual cortical activity, may alter sensorimotor control by modulating visual information processing. However, it is unclear whether tSMS over the visual cortex alters dynamic stability and static postural control in individuals with recurrent lateral ankle sprains. Herein, we aimed to investigate the effects of a single dose of tSMS placed over the visual cortex on dynamic stability and static postural control in individuals with recurrent lateral ankle sprains.

**Methods:**

In a randomized, double-blind placebo-controlled trial, 40 participants with recurrent lateral ankle sprains were randomly allocated to a tSMS or Sham group. During intervention, tSMS or sham stimulation was applied to the visual cortex for 30 min. The outcome measures included time to stabilization in the anteroposterior and mediolateral directions after a unilateral drop landing task and static postural control measured by the clinical test of sensory interaction and balance before and after the intervention.

**Results:**

No significant group-by-time interactions were found in time to stabilization in the anteroposterior and mediolateral directions as well as scores of the clinical test of sensory interaction and balance (*P* > 0.05).

**Conclusion:**

A single session of tSMS applied over the visual cortex did not induce measurable changes in dynamic stability or static postural control in individuals with recurrent lateral ankle sprains. However, because visual processing was not directly assessed following tSMS, it remains unclear whether the tSMS protocol used in this study effectively modulated visual cortical activity.

**Clinical Trial Registration:**

https://www.umin.ac.jp/ctr/, identifier UMIN000052760.

## 1 Introduction

Lateral ankle sprains represent the most prevalent and frequent injury with a high rate of recurrence (1). Over half of individuals with a history of lateral ankle sprain have experienced a recurrent injury (1). Individuals with recurrent lateral ankle sprains have reduced physical activity levels (2), poor health-related quality of life (3), as well as potentially increased risk of falls and posttraumatic ankle osteoarthritis (4–6). These negative health concerns in individuals with recurrent lateral ankle sprains suggest the need for further research focused on more effective rehabilitative strategies in these individuals.

Altered reorganizations of sensorimotor system following an initial lateral ankle sprain may lead to altered postural control in individuals with recurrent lateral ankle sprains (7). Somatosensory impairments following an initial lateral ankle sprain may contribute to altered postural control in individuals with recurrent lateral ankle sprains by reducing their capacity to effectively process and integrate somatosensory feedback within the central nervous system (7, 8). If a source of sensory input is impaired, the sensorimotor system reweights the importance of available visual and/or vestibular information to compensate for somatosensory deficits (9–11). However, this compensatory strategy may be insufficient in dynamic or unpredictable environments, ultimately contributing to persistent postural control deficits observed in individuals with recurrent lateral ankle sprains (8, 12).

Individuals with chronic ankle instability (CAI), a pathological condition characterized by recurrent lateral ankle sprains and episodes of ankle “giving-way” (13), may demonstrate more complex and broader motor-behavioral alterations, including triplanar hip strength deficits and altered movement patterns of proximal joints (14–16). The persistence and complexity of motor-behavioral impairments in these individuals, which cannot be fully explained by peripheral factors alone, suggest an additional contribution from central mechanisms (14, 17). Furthermore, recent neurophysiological evidence suggests that these motor-behavioral impairments may be linked to altered central nervous system processing, including cortical hyperactivation (17), for example, increased activation in the prefrontal and visual cortices during a simple motor task in individuals with CAI (18–20). The findings indicate a shift toward visually guided motor strategies rather than automatic somatosensory-based control. Although these neurophysiological alterations have been demonstrated primarily in individuals meeting established CAI criteria, recurrent lateral ankle sprains are recognized as a clinically relevant component within the CAI continuum (21). Therefore, similar motor-behavioral and cortical alterations may also be present in individuals with recurrent lateral ankle sprains, although this hypothesis has not yet been directly confirmed. This pattern of increased cortical activation in the visual-motor processing area may reflect a maladaptive neuroplastic response to recurrent injury, resulting in inefficient sensorimotor integration (7, 17). These neurophysiological alterations may compromise the ability to flexibly adjust motor behavior under varying sensory demands, particularly during dynamic tasks such as single-leg landing tasks (22).

Recently, neuromodulation techniques, such as transcranial direct current stimulation, have been applied in individuals with CAI to improve postural control and stability; however, findings across studies have been inconsistent (23–25). Efficient postural control is considered to rely on more automatic sensorimotor processes, which are characterized by reduced cortical involvement (26). This raises the possibility that experimentally reducing visual cortical activity could help clarify the role of visual processing in postural control and sensory reweighting. Transcranial static magnetic stimulation (tSMS) is a non-invasive brain stimulation that reduces neural activity at a specific cortical area by placing a neodymium magnet on the human scalp (27). tSMS over the visual cortex increases occipital alpha activity, indicating reduced neural excitability in the visual cortex, and slows visual search performance and cognition during tSMS (28). By selectively reducing the visual cortex activity (27, 28), tSMS may serve as a tool to reveal the extent of visual dependence and to examine how the central nervous system reorganizes sensorimotor strategies when visual processing is disrupted. Understanding these neurophysiological mechanisms could provide insights into the development of targeted neuromodulatory interventions to restore more balanced sensorimotor integration in individuals with recurrent lateral ankle sprains. This knowledge gap limits our ability to design neural-targeted approaches that promote optimal sensorimotor function. Therefore, our primary aim in this study was to investigate the effects of a single session of tSMS over the visual cortex on dynamic stability in individuals with recurrent lateral ankle sprains. Secondarily, we aimed to investigate the acute effects of tSMS on static postural control in these individuals. In addition, as an exploratory analysis, we examined whether tSMS altered sensory organization strategies during single-leg balance.

Applying tSMS to the visual cortex can temporarily suppress compensatory cortical hyperactivation in that region (28), thereby disrupting the brain’s over-reliance on visual input and promoting re-engagement of alternative sensory systems. Thus, this intervention may have bidirectional effects on postural control in individuals with recurrent lateral ankle sprains. On the one hand, reducing visual dependence may enhance sensorimotor integration by forcing the central nervous system to upweight and better utilize somatosensory information, potentially improving postural stability and control (29). Conversely, individuals with recurrent lateral ankle sprains demonstrate impaired somatosensory processing, which may compromise their ability to compensate effectively when visual input is reduced (30). In such cases, tSMS may temporarily diminish postural control because of insufficient reweighting of alternative sensory inputs. Based on the proposed role of visual cortical excitability in sensory reweighting, we considered two competing hypotheses. First, a single session of tSMS applied over the visual cortex would improve dynamic stability and static postural control in individuals with recurrent lateral ankle sprains by promoting greater reliance on somatosensory information. Alternatively, if suppression of visual cortical excitability disrupted sensory integration, tSMS would impair dynamic stability and static postural control in these individuals.

## 2 Methods

### 2.1 Study design

We used a double-blinded, randomized placebo-controlled trial design. Participants were randomly allocated into a tSMS or sham group (Figure 1). A single investigator, who is responsible for collecting and analyzing data, was blinded to group membership. Participants were also blinded to their group membership. The primary outcomes were dynamic stability measures assessed by time to stabilization (TTS) in the anteroposterior and mediolateral directions. Static postural control measures, including the condition scores and composite score obtained from the clinical test of sensory interaction and balance (CTSIB), were defined as secondary outcomes. In addition, the somatosensory, visual, vestibular, and visual preference ratios derived from the CTSIB were analyzed as exploratory outcomes. All study protocols were approved by the Ethics Review Committee for Medical and Health Research Involving Human Subjects at Ritsumeikan University (BKC-LSMH-2023-008). The current study has been registered in a clinical trials registry (registration number UMIN000052760).

**Figure 1:**
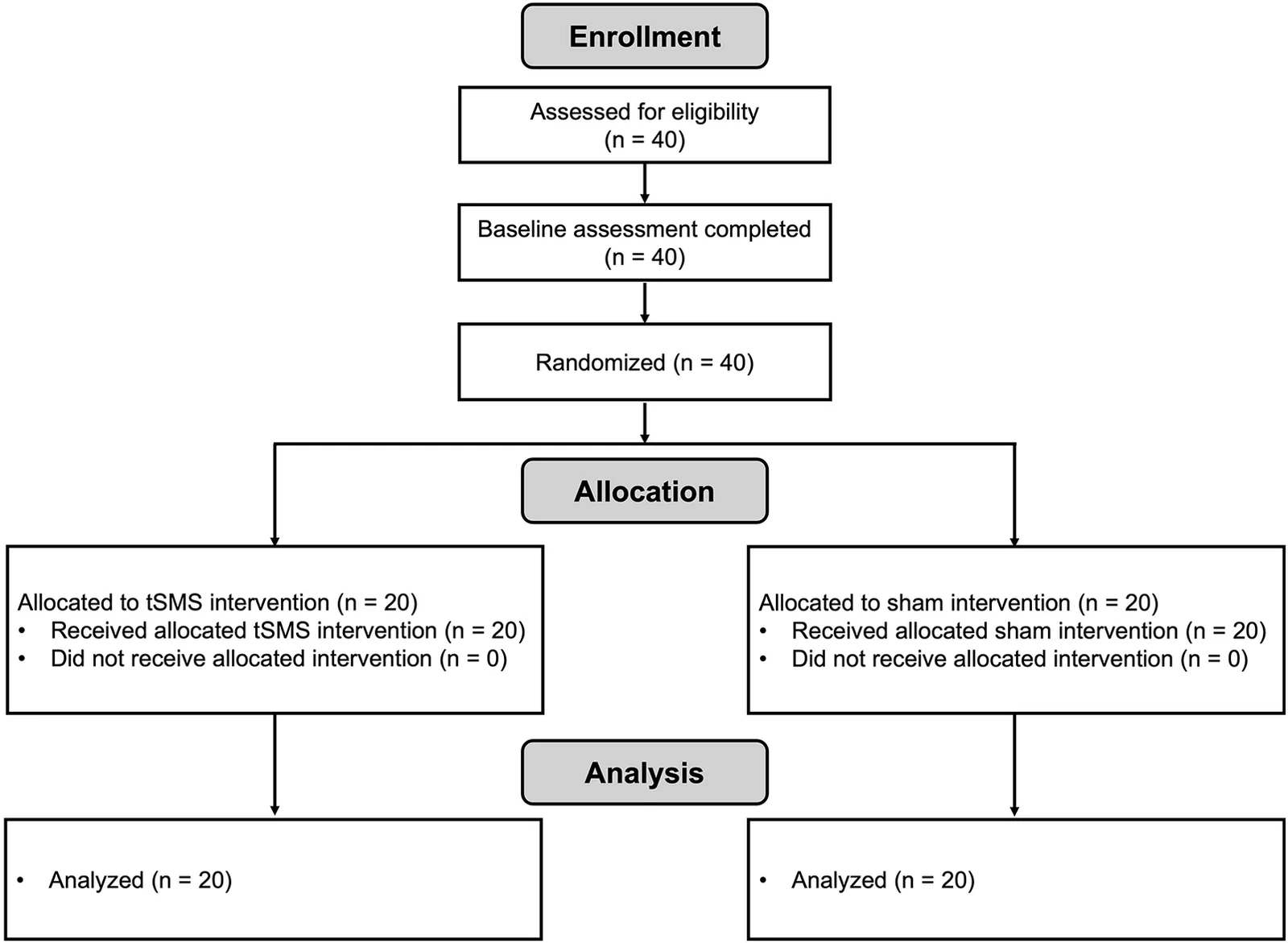
A flowchart: All participants were equally assigned to either the transcranial static magnetic stimulation (tSMS) group or the sham group.

### 2.2 Participants

Forty participants with recurrent lateral ankle sprains were recruited between November 2023 and August 2024 from the university community and volunteered for this study (Table 1). All participants read and signed informed written consent approved by the Ethics Review Committee for Medical and Health Research Involving Human Subjects. All participants had a history of lateral ankle sprain that caused swelling, pain, and temporary loss of function for at least 1 day (13). An additional criterion was a history of two or more lateral ankle sprains (31). Participants were recruited based on a history of recurrent lateral ankle sprains and were not required to meet established criteria for CAI, including the self-reported ankle instability and episodes of giving-way. The Identification of Functional Ankle Instability (IdFAI) and the Cumberland Ankle Instability Tool (CAIT) were administered to characterize the level of self-reported ankle instability in the study population rather than to determine study eligibility. All participants had no history of diagnosed balance or vestibular disorders, self-reported low back pain in the previous 6 months, surgery or fracture in the lower extremity, history of concussion in the past 6 months, or any self-reported musculoskeletal or neurovascular injuries and disorders in the chest and the lower extremity other than lateral ankle sprains in the previous 2 years. In addition, participants were excluded if they had pacemakers, brain stimulators, medication pumps, or any type of metal object that might influence a physical threat during tSMS. If a participant had a history of bilateral ankle injury, we used the limb with the greater number of lateral ankle sprains and higher degree of self-reported instability employing IdFAI and CAIT (13).

**Table 1.** Anthropometric and ankle injury characteristics at baseline.

Variable	tSMS		Sham		*P* value
	Mean ± SD	Median [IQR]	Mean ± SD	Median [IQR]	
n	20 (10 males, 10 females)		20 (10 males, 10 females)		
Age (year)	21.3 ± 1.5	21.0 [20.0–22.0]	22.0 ± 2.1	21.0 [20.8–24.0]	0.355
Height (cm)	165.8 ± 7.9	167.5 [158.5–171.1]	166.6 ± 8.5	169.0 [159.3–172.8]	0.757
Body mass (kg)	58.0 ± 9.1	59.7 [54.5–69.0]	61.3 ± 8.5	59.7 [54.5–69.0]	0.234
Body mass index (kg/m^2^)	21.0 ± 2.2	21.6 [20.9–23.6]	22.0 ± 1.7	21.6 [20.9–23.6]	0.105
Number of lateral ankle sprains	4.1 ± 2.4 (Min: 2, Max: 20)	3.5 [2.0–5.3]	4.0 ± 4.1 (Min: 2, Max: 12)	3.0 [2.0–3.3]	0.289
Time since last ankle sprain (month)	34.8 ± 24.5	34.5 [11.5–51.0]	46.0 ± 37.5	36.0 [15.8–72.0]	0.529
Number of giving-way episodes in past 6 month	2.0 ± 2.4 (Min: 0, Max: 10)	1.5 [0.8–2.3]	2.0 ± 3.0 (Min: 0, Max: 12)	1.0 [0.0–2.3]	0.461
Identification of Functional Ankle Instability	12.7 ± 5.6	13.0 [8.8–17.0]	12.1 ± 7.0	11.5 [5.0–18.0]	0.738
Cumberland Ankle Instability Tool	23.7 ± 4.1	24.0 [22.3–27.0]	22.9 ± 5.1	24.0 [19.8–26.5]	0.587

IQR, interquartile range, SD, standard deviation, tSMS, transcranial static magnetic stimulation.

### 2.3 Sample size calculation

Because no previous studies have investigated the effects of tSMS on postural control in individuals with recurrent lateral ankle sprains, the sample size was determined using data from a previous study that applied a comparable non-invasive brain stimulation method (transcranial direct current stimulation) in this population (23). The calculation was based on a postural control outcome, specifically TTS, and was designed to detect a group-by-time interaction using a two-way repeated-measures ANOVA. Although the previous study reported a large effect size following a 6-week intervention (Cohen’s *f* = 0.51), based on a significant group-by-time interaction (*η*_p_² = 0.209) (23), a medium effect size (Cohen’s *f* = 0.25) was used here for a more conservative estimate, as this study assessed the immediate effects of a single-session intervention. With a predetermined alpha level of 0.05 and a statistical power of 0.80, the analysis indicated that 17 participants per group were required. To ensure sufficient analyzable data in the presence of potential missing measurements due to technical issues, 20 participants were recruited for each group, resulting in a total of 40 participants.

### 2.4 Experimental procedures

Participants reported to the research laboratory for two sessions: an initial screening session to determine study eligibility, followed by a second session that included intervention as well as pre- and post-intervention assessments of dynamic stability and static postural control. The average interval between sessions was 17.3 days, during which no participant sustained injuries. During the pre-intervention assessment, the participants performed a unilateral drop landing task and the CTSIB during single-leg stance if their leg was involved in recurrent lateral ankle sprains. After the pre-intrevention assessment, the participants were seated in an armchair for 30 min while magnetic or sham stimulation was placed on the scalp over the visual cortex. The participants were randomly assigned to the tSMS or Sham group using a computer-generated random sequence stratified by sex, age, number of previous lateral ankle sprains, and level of self-reported ankle instability. Allocation was implemented through sequentially numbered, opaque, sealed envelopes prepared by a single investigator who was not involved in data collection or analysis. After the end of the stimulation, the participants performed the unilateral drop landing task and the CTSIB in their involved leg as the post-intervention assessment. To assess the success of participant blinding, all participants were asked at the end of the study to guess which intervention they had received (tSMS, sham stimulation, or “unclear”). The post-intervention assessments were completed within 30 min, as the effects of tSMS on cortical excitability last approximately 30 min (32). The order of dynamic and static balance tasks was randomized across participants and between pre- and post-intervention assessments.

### 2.5 Intervention

The participants were seated in an armchair to keep relaxed during the intervention (Figure 2). For tSMS, a cylindrical nickel-plated (Ni–Cu–Ni) NdFeB magnet with a 50 mm diameter and 30 mm thickness (Model N-50; NeoMag, Chiba, Japan) was placed on the scalp over the visual cortex for 30 min by an arm-type light stand (C-stand; Avenger, Cassola, Italy). The duration of the intervention was established based on a previous study (32). The representational area of the visual cortex was determined by the location at Oz in the 10–20 electroencephalography system (28). The maximum energy density was 406 kJ/m^3^, with a nominal strength of 863 N (33). The surface magnetic flux density was approximately 5340 G (33). A non-magnetic stainless-steel cylinder of the same size, mass, and appearance as the NdFeB magnet was used for the Sham group. All participants were instructed to open their eyes, maintain full contact between their soles and the floor, and refrain from sleeping and talking during the intervention. Adverse events were monitored throughout the intervention and during the post-intervention assessment and defined as any unintended physical or psychological response including, but not limited to, the following: discomfort, headache, blurred vision, dizziness, nausea, mood changes, or any other unexpected symptoms. The participants were asked about these symptoms immediately after the intervention and again before leaving the laboratory.

**Figure 2:**
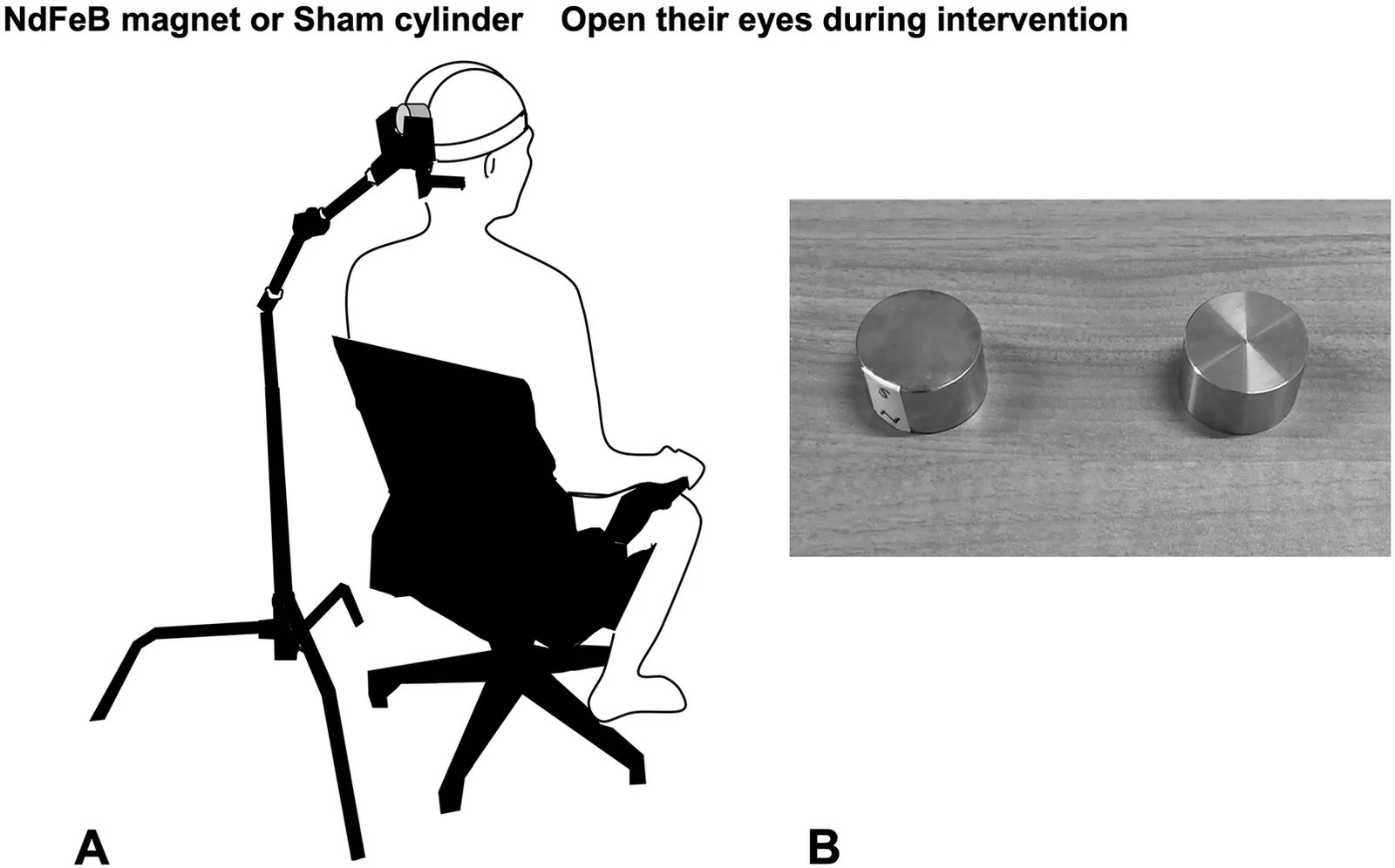
An experimental setup during the intervention. **(A)** Participants were required to keep relaxed and open their eyes in a seated position during the intervention. **(B)** The NdFeB magnet (left) and the non-magnetic stainless-steel cylinder (right) were settled.

### 2.6 Dynamic stability assessment

Dynamic postural stability was assessed using a unilateral drop landing task previously described (34) and modified for this study. The participants were instructed to stand barefoot on their involved leg on a 31 cm-high box, positioned 30 cm away from the center of the 11-cm-high force platform (SS-FP40AO-SY, Sports Sensing Co., Ltd., Fukuoka, Japan), creating a total drop height of 20 cm, with their toes at the edge. For the unilateral drop landing task, the participants were asked to jump off the testing leg toward the force platform, land with the testing leg on the center of the force platform with their arms folded across their chest throughout the drop landing task, and quickly assume a single-leg stance while looking straight ahead at a target placed 3.50 m away from the force plate and 1.70 m above the floor. The participants were required to maintain this position for 8 s with their arms folded in front of their chest. They underwent three practice trials to familiarize themselves with the task and then completed three testing trials. A trial was discarded and repeated if the participants failed to maintain a single-leg stance for 8 s and removed the testing limb from the force platform; they hopped or took a step with the testing limb; the non-testing limb made contact with the force platform or the testing limb; they used a double-leg landing strategy to gain stability; or they removed their hands from their chest before completing the task for 8 s.

Ground reaction force data were quantified using the Dynamic Balance Assessment System software integrated with the force platform (version SS-FPSW01, Sports Sensing Inc., Fukuoka, Japan). The ground reaction force data were sampled at 1,000 Hz and low-pass-filtered using a zero-time-shift, second-order Butterworth filter with a cutoff frequency of 70 Hz (35). Initial contact with the force platform was identified with greater than 10 N of the vertical component of the ground reaction force. TTS was calculated using a custom-written MATLAB software program (MathWorks, Inc.) referred by previously described methods (36). In this study, a normalized reference variable was calculated by identifying the ground reaction force ranges in both the anteroposterior and the mediolateral directions during the last 1 s of each trial. These ground reaction force data were then normalized by dividing by each participant’s body weight. The averaged normalized reference variable and the standard deviations from the three trials were calculated, and a normalized range-of-variation variable was determined by adding three standard deviations to the overall mean range for each direction. An unbounded third-order polynomial was applied to the rectified force data during more than 5 s following landing for each participant and trial in both directions. To obtain the range-of-variation value for each participant, the normalized range-of-variation variable was multiplied by their body weight in Newtons. TTS was identified as the time point where the unbounded third-order polynomial crossed the range-of-variation value (36). The averaged values of TTS from the three trials were used for statistical analysis.

### 2.7 Clinical test of sensory interaction and balance

Static postural control was assessed by using the CTSIB, comprising six conditions created by combining three visual conditions (eyes-open, eyes-closed, and the visual-conflict conditions) with two surface conditions (firm and foam) (Figure 3) (37). For each condition, the participants completed three barefoot single-leg stance trials while placing their hands on their waists. They wore the visual-conflict dome to create a sway-referenced visual input (Conditions 3 and 6). Under Conditions 4, 5, and 6, the participants stood on a sway-referenced surface using the foam pad (AIREX® balance pad; AIREX, Sins, Switzerland) to reduce the reliability of somatosensory input. The testing order of condition was randomized for each participant and each time point. The participants were instructed to maintain balance as much as possible with a maximum duration of 30 s. A single investigator stopped the time if the participants shifted or slid their testing foot; the non-testing limb touched the surface or the testing limb; they moved their hands away from their waists; or they opened their eyes during the eyes-closed conditions (Conditions 2 and 5). The average time was calculated from the three trials for each condition. The composite score was calculated as the mean of the average time across the six conditions. Somatosensory, visual, and vestibular ratios were calculated by dividing the mean time in Conditions 2, 4, or 5 relative to Condition 1, respectively, and expressed as percentages (37). These ratios reflect the ability to utilize somatosensory, visual, and vestibular inputs, respectively. The preference ratio was calculated to quantify reliance on inaccurate visual information relative to absent vision [(Condition 3 + Condition 6) ÷ (Condition 2 + Condition 5) × 100], which reflect the tendency to rely on visual information, with higher values indicating the greater dependence on visual inputs (37).

**Figure 3:**
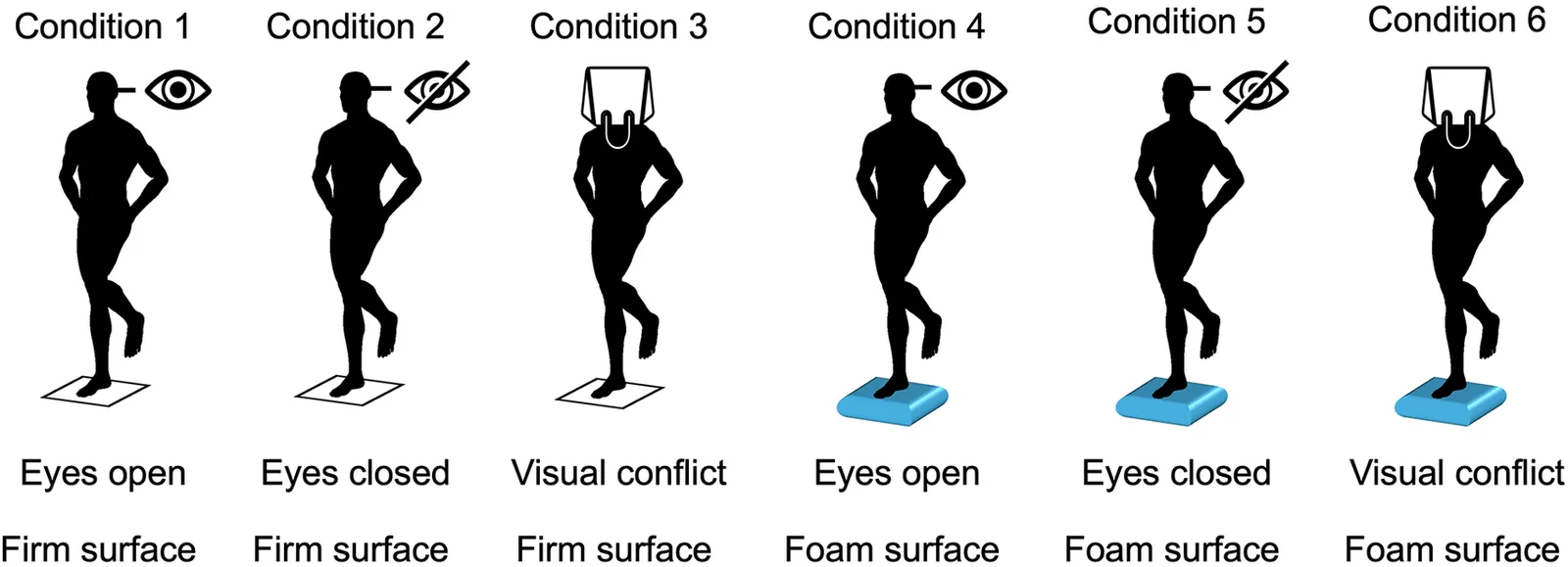
Six conditions for the clinical test of sensory interaction and balance during a single-leg stance. The figure was created by the authors using Magic Poser (Wombat Studio, Inc.).

### 2.8 Statistical analysis

Independent *t*-tests or the Mann–Whitney *U* test were used to compare anthropometric variable, ankle injury characteristics, the IdFAI and CAIT scores, and pre-intervention outcome measures between groups, depending on the results of the Shapiro–Wilk normality test. Normality was assessed using the Shapiro–Wilk test for each group at each time point, and residual Q–Q plots were visually inspected. Although some outcome variables deviated from normality, the primary analyses were conducted using two-way repeated-measures (group × time) ANOVAs because of the balanced design with equal group sizes. As only two repeated measurements were included, the assumption of sphericity was inherently satisfied. No multiplicity adjustment was performed across the secondary and exploratory CTSIB outcomes because these analyses were considered exploratory. Bonferroni-adjusted *post hoc* comparisons were performed when a statistically significant interaction effect was observed. In addition, between-group treatment effects were estimated as the difference in mean change from pre- to post-intervention assessment between the tSMS and Sham groups, with corresponding 95% confidence intervals (CIs). Positive values indicate greater changes in the tSMS group compared with the Sham group, whereas negative values indicate greater changes in the Sham group than the tSMS group. To assess the robustness of the findings, sensitivity analyses using two-way (group × time) linear mixed-effects models were additionally performed, with participants included as a random effect. Fisher's exact test was used to examine the association between actual group allocation and participant guesses. Significance was set *a priori* at *P* < 0.05 using SPSS 29.0 (IBM Corp., Armonk, NY, USA). Effect sizes were expressed as partial eta squared (*η*_p_^2^) interpreted as small (0.01 ≤ *η*_p_^2^ < 0.06), medium (0.06 ≤ *η*_p_^2^ < 0.14) or large (*η*_p_^2^ ≥ 0.14). In addition, Cohen's *d* effect size and 95% CIs were calculated for each pairwise comparison to facilitate interpretation of the magnitude of differences in each outcome measure (38). The strength of the effect sizes was interpreted as small (*d* < 0.40), medium (0.40 ≤ *d* < 0.80), or large (*d* ≥ 0.80) (38).

## 3 Results

At baseline, no significant between-group differences were observed in anthropometric characteristics, ankle injury characteristics, IdFAI and CAIT scores (*P* >0.050, Table 1), or pre-intervention outcome measures (*P* >0.050, Table 2). There was no adverse event in this study. In the tSMS group, 13 participants (65%) reported that they were unsure which intervention they had received, six (30%) correctly guessed tSMS, and one (5%) guessed sham stimulation. In the sham group, 12 participants (60%) reported being unsure, five (25%) correctly guessed sham stimulation, and three (15%) guessed tSMS. There was no significant association between actual group allocation and participant guesses (*P* = 0.233).

**Table 2.** Outcome measures at the pre-intervention assessment.

Variable	tSMS		Sham		*P* value
	Mean ± SD	Median [IQR]	Mean ± SD	Median [IQR]	
TTS-AP (s)	1.44 ±1.14	1.46 [1.43–1.56]	1.47 ± 1.52	1.45 [1.37–1.52]	0.157
TTS-ML (s)	1.67 ± 0.72	1.57 [1.36–1.96]	1.83 ± 0.70	1.63 [1.39–2.01]	0.429
Condition 1 (s)	29.2 ± 3.4	30.0 [30.0–30.0]	29.9 ± 0.4	30.0 [30.0–30.0]	0.799
Condition 2 (s)	22.5 ± 9.6	28.3 [18.0–30.0]	25.3 ± 4.8	26.0 [21.1–30.0]	0.841
Condition 3 (s)	22.0 ± 11.1	29.9 [11.4–30.0]	22.2 ± 7.5	22.5 [18.0–30.0]	0.547
Condition 4 (s)	23.9 ± 8.5	28.3 [21.0–30.0]	27.8 ± 3.5	30.0 [25.9–30.0]	0.201
Condition 5 (s)	5.7 ± 3.2	5.3 [3.6–6.3]	5.7 ± 3.0	5.2 [3.4–7.3]	0.862
Condition 6 (s)	5.0 ± 3.1	4.2 [3.3–5.6]	4.9 ± 2.6	4.0 [3.3–5.2]	0.989
Composite score (s)	18.4 ± 4.6	20.9 [15.8–21.4]	19.0 ± 3.7	19.2 [18.2–21.3]	0.820
Somatosensory ratio (%)	76.1 ± 30.3	94.3 [59.9–100.0]	84.5 ± 16.2	88.9 [70.2–100.0]	0.862
Visual ratio (%)	80.2 ± 27.4	94.3 [69.9–100.0]	93.0 ± 12.0	100.0 [86.4–100.0]	0.157
Vestibular ratio (%)	19.4 ± 10.4	17.8 [13.4–21.0]	19.0 ± 10.0	17.4 [11.3–24.3]	1.000
Preference ratio (%)	99.5 ± 42.5	93.6 [82.5–106.7]	87.2 ± 20.5	91.8 [76.1–102.3]	0.512

IQR, interquartile range, SD, standard deviation, tSMS, transcranial static magnetic stimulation; TTS, time to stabilization; AP, anteroposterior; ML, mediolateral.

No group × time interactions were found for TTS in either anteroposterior or mediolateral directions with small effect sizes (*P* > 0.050, *η*_p_^2^ < 0.060; Table 3). There were small effect sizes for TTS measures between the tSMS and the sham groups at post-intervention, with 95% CIs overlapping zero (Figure 4).

**Table 3:** Pre-test and post-test time to stabilization (TTS) (mean ± standard deviation).

Variable	tSMS		Sham					
	Pre-test	Post-test	Pre-test	Post-test	Group × Time Interaction *P* value (partial *η*^2^)	Group main effect *P*-value (partial *η*^2^)	Time main effect *P*-value (partial *η*^2^)	Between-group difference in changes (95% CI)
TTS-AP (s)	1.44 ± 1.14	1.44 ± 1.24	1.47 ± 1.52	1.46 ± 1.51	0.856 (0.001)	0.520 (0.011)	0.902 (0.0004)	−0.01 (−0.17, 0.10)
TTS-ML (s)	1.67 ± 0.72	1.87 ± 0.93	1.83 ± 0.70	1.63 ± 0.58	0.159 (0.052)	0.821 (0.001)	0.991 (0.000003)	−0.11 (−0.69, 0.47)

Between-group difference in changes was estimated as the difference in mean change from pre- to post-intervention assessment between the tSMS and the Sham groups.

tSMS, transcranial static magnetic stimulation; AP, anteroposterior; ML, mediolateral; CI, confidence intervals.

**Figure 4:**
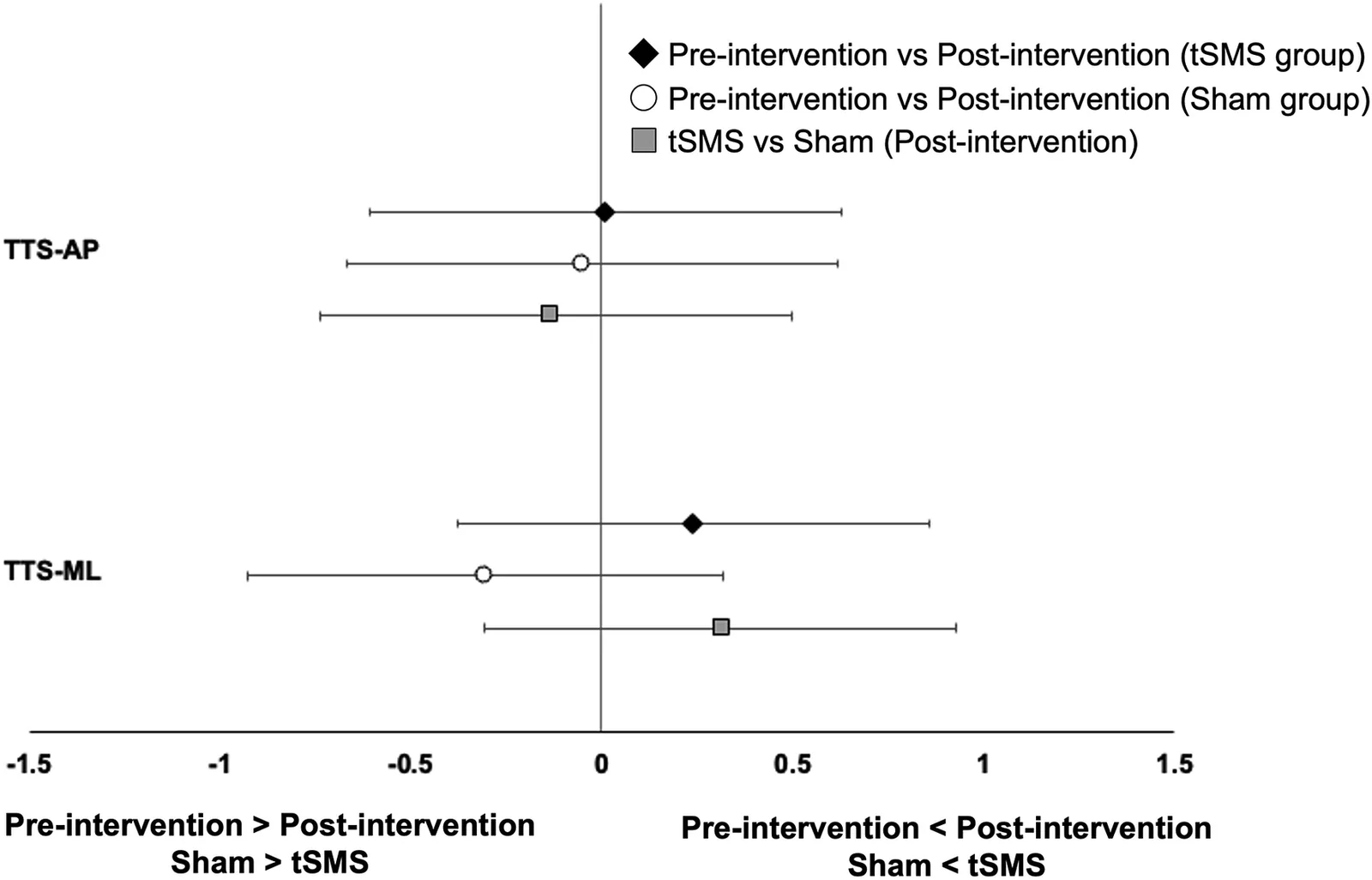
Cohen's *d* effect sizes and associated 95% confidence intervals for time to stabilization (TTS). tSMS, transcranial static magnetic stimulation; AP, anteroposterior; ML, mediolateral.

There were no group × time interactions for mean times in all conditions, the composite scores, as well as somatosensory, visual, vestibular, and preference ratios of the CTSIB with small to medium effect sizes (*P* > 0.050, *η*_p_^2^ ≤ 0.070; Table 4). Significant group main effects were observed for Condition 4 (*P* = 0.040, *η*_p_^2^ = 0.106) and visual ratio (*P* = 0.037, *η*_p_^2^ = 0.110) with medium effect sizes (Table 4). Furthermore, significant time main effects were found for Condition 3, Condition 4, Condition 5, Condition 6, the composite scores, as well as visual and vestibular ratios with medium to large effect sizes (*P* < 0.050, 0.131 ≤ *η*_p_^2^ ≤ 0.471; Table 4). However, several exploratory pairwise comparisons demonstrated medium effect sizes, particularly for Condition 4, Condition 5, and sensory ratio measures (Figure 5).

**Table 4:** Pre-test and post-test clinical test of sensory interaction and balance scores (mean ± standard deviation).

	tSMS		Sham					
Variable	Pre-test	Post-test	Pre-test	Post-test	Group × Time Interaction *P*-value (partial *η*^2^)	Group main effect *P*-value (partial *η*^2^)	Time main effect * P*-value (partial *η*^2^)	Between-group difference in changes (95% CI)
Condition 1 (s)	29.2 ± 3.4	29.0 ± 2.9	29.9 ± 0.4	29.8 ± 0.7	0.810 (0.002)	0.296 (0.029)	0.407 (0.018)	−0.38 (−1.12, 0.36)
Condition 2 (s)	22.5 ± 9.6	24.7 ± 8.3	25.3 ± 4.8	26.3 ± 5.8	0.567 (0.009)	0.305 (0.028)	0.133 (0.059)	2.31 (−1.83, 6.44)
Condition 3 (s)	22.0 ± 11.1	22.9 ± 9.5	22.2 ± 7.5	26.3 ± 5.0	0.139 (0.057)	0.488 (0.013)	0.022 (0.131)	−0.38 (−4.69, 3.92)
Condition 4 (s)	23.9 ± 8.5	25.6 ± 8.6	27.8 ± 3.5	29.7 ± 0.8	0.916 (0.0002)	0.040 (0.106)	0.011 (0.158)	0.80 (−1.91, 3.50)
Condition 5 (s)	5.7 ± 3.2	8.2 ± 4.2	5.7 ± 3.0	8.2 ± 6.5	0.972 (0.00003)	0.987 (0.00001)	<0.001 (0.282)	−1.43 (−4.02, 1.16)
Condition 6 (s)	5.0 ± 3.1	6.5 ± 3.0	4.9 ± 2.6	7.7 ± 3.6	0.288 (0.030)	0.455 (0.015)	0.001 (0.248)	−0.44 (−2.94, 2.06)
Composite score (s)	18.4 ± 4.6	20.0 ± 4.8	19.0 ± 3.7	20.9 ± 3.2	0.538 (0.010)	0.555 (0.009)	<0.001 (0.471)	0.08 (−1.13, 1.29)
Somatosensory ratio (%)	76.1 ± 30.3	83.5 ± 25.3	84.5 ± 16.2	87.9 ± 18.8	0.562 (0.009)	0.328 (0.025)	0.126 (0.060)	8.49 (−5.22, 22.20)
Visual ratio (%)	80.2 ± 27.4	86.8 ± 27.4	93.0 ± 12.0	99.7 ± 4.0	0.995 (0.000001)	0.037 (0.110)	0.011 (0.159)	3.32 (−6.61, 13.25)
Vestibular ratio (%)	19.4 ± 10.4	27.8 ± 13.5	19.0 ± 10.0	27.7 ± 21.6	0.960 (0.0001)	0.945 (0.0001)	<0.001 (0.285)	−4.55 (−13.27, 4.17)
Preference ratio (%)	99.5 ± 42.5	90.5 ± 26.1	87.2 ± 20.5	103.9 ± 33.6	0.099 (0.070)	0.929 (0.0002)	0.615 (0.007)	−20.65 (−51.71, 10.41)

Between-group difference in changes was estimated as the difference in mean change from pre- to post-intervention assessment between the tSMS and the Sham groups.

tSMS, transcranial static magnetic stimulation; CI, confidence intervals.

**Figure 5:**
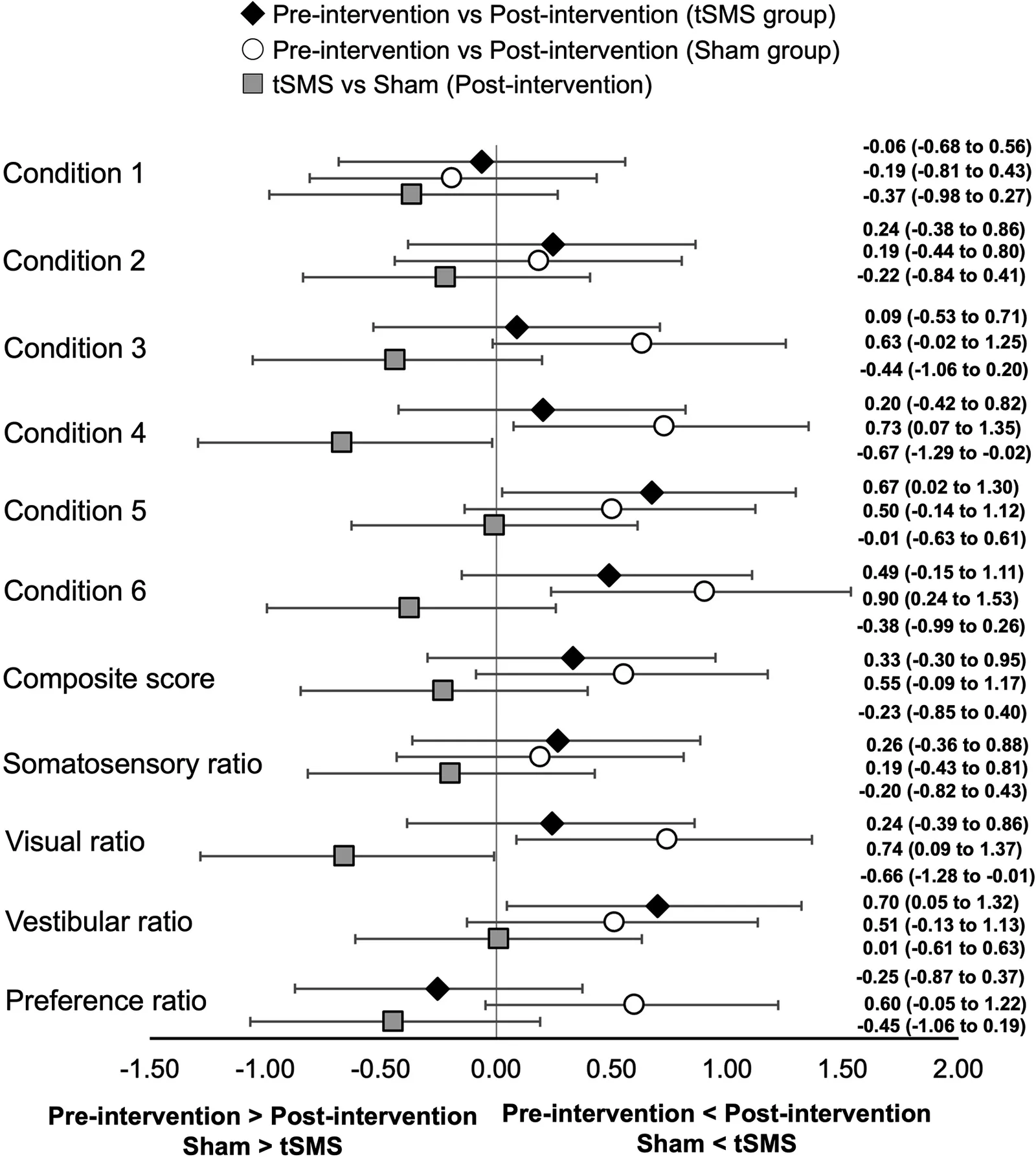
Cohen's *d* effect sizes and associated 95% confidence intervals for the clinical test of sensory interaction and balance scores, as well as somatosensory, visual, vestibular, and preferece ratios. tSMS, transcranial static magnetic stimulation**.**

Sensitivity analyses using linear mixed-effects models yielded results consistent with the primary analyses. Specifically, no significant group × time interactions were observed for TTS measures or any CTSIB-derived measures (*P* > 0.050). For CTSIB measures, the linear mixed-effects models identified significant group main effects for Condition 4 (*P* = 0.040) and the visual ratio (*P* = 0.037), as well as significant time main effects for Condition 3 (*P* = 0.022), Condition 4 (*P* = 0.011), Condition 5 (*P* < 0.001), Condition 6 (*P* = 0.001), composite scores (*P* < 0.001), and the visual (*P* = 0.011) and vestibular ratios (*P* < 0.001).

## 4 Discussion

A single session of tSMS placed over the visual cortex region was not associated with significant changes in dynamic stability or static postural control in the current study. Contrary to our primary hypothesis, no improvement in postural control was observed following tSMS. Likewise, the alternative hypothesis that suppression of visual cortical excitability would impair postural control was also not supported. Importantly, because visual cortical activity or excitability was not directly measured, target engagement could not be verified. Therefore, these findings should be interpreted with caution. Previous studies have shown that 10 min of tSMS placed over the visual cortex reduces visual cortical excitability and influences visual search performance (28), and that a 30-min application may induce stronger and longer-lasting suppression of cortical excitability than a 10-min application (32). Based on these findings, we selected the present tSMS protocol. Nevertheless, because no neurophysiological or behavioral measures of visual cortical function were obtained, it remains unclear whether the tSMS protocol used in this study successfully modulated visual cortical excitability. Therefore, the absence of measurable changes in dynamic stability and static postural control may reflect either that any tSMS-induced modulation of visual cortical excitability, if present, was insufficient to alter postural control or that the tSMS protocol designed for this study did not achieve sufficient target engagement. Future studies incorporating neurophysiological measures (e.g., EEG) and behavioral assessments of visual function are needed to verify target engagement and to clarify the role of visual cortical modulation in postural control in individuals with recurrent lateral ankle sprains.

A single tSMS session placed over the visual cortex did not result in differential changes in TTS in anteroposterior and mediolateral directions compared with sham stimulation in participants with recurrent lateral ankle sprains. If tSMS effectively modulated visual processing during the dynamic stability assessment, one possible interpretation is that the participants maintained dynamic stability through compensatory sensorimotor mechanisms such as sensory reweighting and/or feedforward motor control. However, these mechanisms were not directly assessed in the current study, and this interpretation therefore remains speculative.

Our findings may be considered in the context of previous research examining the effects of altered visual focus on landing mechanics and dynamic stability (39). Terada et al. found no differences in kinematics or dynamic stability during unilateral drop landing tasks between looking-up and looking-down conditions in individuals with CAI (39). The authors proposed that this lack of effect of altered visual input might reflect adaptation within the central nervous system through sensorimotor system reorganization and sensory reweighting (39). Similarly, if the current tSMS intervention altered visual processing, the participants may have maintained dynamic stability by increasing reliance on other available sensory inputs or feedforward motor control. However, because neither target engagement nor these compensatory mechanisms was directly assessed, this explanation remains speculative. Further research incorporating direct assessments of visual processing, sensory reweighting, and feedforward control is required.

The CTSIB scores in all conditions did not differ significantly between the tSMS and the sham stimulation groups. Thus, our findings did not demonstrate significant intervention-related changes in static postural control following the tSMS application to the visual cortex in individuals with recurrent lateral ankle sprains. A single session of tSMS over the supplementary motor area has been reported to shorten the electromyogram latency difference between a prime mover muscle and a postural muscle during a rapid shoulder flexion task performed in an upright bilateral stance in young healthy adults (40). However, center-of-pressure parameters remained unchanged following tSMS during the shoulder flexion task (40). These findings suggest that young healthy individuals may maintain postural stability even when neuromuscular and postural control strategies are altered following tSMS (40).

Previous studies suggest that individuals with CAI exhibit rigid hip strategies to stabilize their posture during static unilateral stance tasks compared with healthy individuals (15, 16). Taken together, if visual processing was altered by tSMS in this study, one possible interpretation is that the participants maintained static postural control through compensatory neuromuscular or sensorimotor strategies (15, 16). However, because we did not confirm whether tSMS altered visual processing or directly assess such compensatory strategies, this interpretation remains speculative. In addition, a potential ceiling effect may partly explain the lack of measurable changes in CTSIB outcomes. CTSIB scores in several conditions, particularly Conditions 1–4, approached the 30-s maximum. This potential ceiling effect may have reduced the sensitivity of the CTSIB to detect intervention-related changes.

Significant main effects of time were observed for the CTSIB scores in Conditions 3–6. These changes from pre- to post-intervention in both groups may reflect learning, familiarization, or repeated-testing effects, rather than changes in postural control induced by tSMS. Although a familiarization session was performed before data collection, and the testing order of the CTSIB conditions was randomized for each participant, our participants in both groups may adapt their postural strategies with repeated CTSIB measurements, particularly under conditions varying sensory demands during single-leg stance. Future studies should incorporate additional familiarization trials or alternative study designs to better distinguish intervention effects from repeated-testing effects.

In exploratory analyses of sensory organization strategies during single-leg stance, no significant group × time interactions were observed. Although medium between-group effect sizes were observed for Condition 4 and the visual ratio between the tSMS and the sham groups at post-intervention, these findings should be interpreted with caution because significant group main effects were detected for these variables. Because these variables did not demonstrate significant group × time interactions, the observed group main effects cannot be attributed to the tSMS intervention.

Furthermore, because multiple outcome measures were analyzed without adjustment for multiple comparisons, these significant group main effects may represent chance findings rather than intervention-related effects. Their clinical and neurophysiological relevance therefore remains uncertain. Therefore, it remains unclear whether tSMS placed over the visual cortex influenced sensory reweighting during postural control. These exploratory findings require confirmation in future studies with larger sample sizes.

The absence of changes in static postural control and sensory organization strategies following tSMS may be attributed to variability in participants’ baseline levels of static postural control and/or sensory organization strategies (7). Individuals with recurrent lateral ankle sprains may exhibit different combinations and degrees of pathomechanical, sensory-perceptual, and motor-behavioral impairments (7). The heterogeneity of this injury suggests that altered sensorimotor control such as visually guided motor behavior may not have been a significant factor for all participants. Similarly, previous research has shown individual variability in responses to neuromodulation, with 52% of participants showing long-term potentiation-like increases in motor-evoked potential amplitudes, while 48% exhibited long-term depression-like decreases after paired associative stimulation (41). Muller-Dahlhaus et al. (41) suggested that such interindividual variability may be related to baseline cortical excitability. Therefore, individual differences in baseline sensorimotor control strategies and cortical excitability may explain the lack of changes in static postural control measures in our recurrent lateral ankle sprains population. Future studies should include a more comprehensive assessment of baseline cortical excitability and sensorimotor function to better understand individual variations in postural control response to tSMS for developing individualized interventions aimed at restoring sensorimotor control in individuals with recurrent lateral ankle sprains.

A single session of tSMS might not be sufficient to influence dynamic stability or static postural control in participants with recurrent lateral ankle sprains. A previous systematic review suggests that a single session of tSMS can modulate cortical excitability as well as certain behavioral outcomes (42). Specifically, tSMS applied to the visual cortex has been shown to reduce cortical activity and impair visual search performance (28). Based on these findings, we hypothesized that a single session of tSMS might influence postural control by modulating cortical processes related to sensorimotor integration. However, contrary to this hypothesis, no measurable changes in dynamic stability or static postural control were observed in the present study.

A possible methodological factor contributing to the absence of measurable changes in postural control relates to the timing of the post-intervention assessments. All post-intervention assessments were initiated immediately after stimulation and completed within 30 min, based on previous reports indicating that the effects of tSMS on cortical excitability persist for approximately 30 min (32, 43). However, because the exact interval between stimulation cessation and each outcome assessment was not recorded, we cannot determine whether potential neuromodulatory effects changed over the course of the assessment period. If tSMS induced a post-effect, assessments performed later in the testing sequence may have captured a smaller effect because of temporal decay. Because the order of outcome assessments was randomized across participants, any potential attenuation would likely have been distributed across outcome measures rather than systematically biasing a particular outcome. Nevertheless, uncertainty regarding the temporal profile of any tSMS post-effects remains. These findings should be interpreted by considering the timing of the post-intervention assessments. Future studies should record the exact timing of each assessment and, where feasible, obtain outcome measures during or immediately after stimulation.

Previous studies have demonstrated that repeated sessions of non-invasive brain stimulation produce more stable and longer-lasting effects on neural excitability and behavioral outcomes compared with a single session (44, 45). However, repeated stimulation does not necessarily result in additive improvements in postural control. For example, anodal transcranial direct current stimulation applied over the motor cortex during a 4-week progressive balance training program did not provide additional benefits for static postural control, neural excitability, or self-reported ankle function compared with sham stimulation in individuals with CAI (25).

Importantly, recent evidence suggests that the effects of neuromodulation may depend on the exercise modality performed during stimulation (25). This perspective indicates that the interaction between stimulation and task demands may be a critical factor in determining behavioral outcomes. Therefore, future studies should examine whether multiple sessions of tSMS, combined with appropriately selected task-specific interventions, can influence postural control and stability in individuals with recurrent lateral ankle sprains.

### 4.1 Limitations

Several methodological limitations should be considered when interpreting the current findings. We did not assess dynamic stability or static postural control in healthy control individuals, which limits direct comparisons to normative levels of these measures.

We did not directly assess visual cortical activity or excitability during or after tSMS using neurophysiological measures such as EEG. We also did not include behavioral assessments of visual function. Consequently, target engagement could not be verified, and we cannot determine whether the current tSMS protocol effectively modulated visual cortical function. Future studies should incorporate neurophysiological and behavioral measures of visual function to verify target engagement and examine the relationship between visual cortical modulation and postural control in individuals with recurrent lateral ankle sprains.

We did not assess dynamic and static postural control during tSMS. A previous study reported the inhibitory effects of tSMS on behavioral performances during tSMS application (28). In our study, we secured a magnet or stainless-steel by the arm-type light stand. Thus, it is difficult to stimulate the visual cortex during postural control because of arising postural sway.

In the current study, the sample size calculation was based on a medium effect size; however, most observed group × time interaction effect sizes were small. Therefore, this study may have been underpowered to detect smaller acute intervention effects of a single session of tSMS on postural control outcomes. The absence of significant group × time interactions should not be interpreted as evidence for the absence of a true effect.

Finally, although participant blinding was assessed, the blinding integrity of the outcome assessor was not formally evaluated. Therefore, the possibility that the outcome assessor's blinding was incomplete cannot be completely excluded.

## 5 Conclusion

A single tSMS session placed over the visual cortex region did not significantly alter TTS, CTSIB scores, and sensory organization strategies compared with sham stimulation in individuals with recurrent lateral ankle sprains. However, because neurophysiological and behavioral measures of visual function were not directly assessed, it remains unclear whether the intervention effectively modulated visual cortical activity or excitability. In addition, because this study was powered to detect a medium effect size, smaller acute intervention effects cannot be excluded. Therefore, our findings should be interpreted within the context of the specific stimulation protocol, participant characteristics, outcome measures, and statistical sensitivity of the current study. Under these experimental conditions, a single session of tSMS did not produce measurable changes in dynamic stability or static postural control; however, these findings should not be interpreted as evidence that modulation of visual cortical excitability has no influence on postural control. Future studies incorporating direct measures of target engagement are needed to clarify the effects of tSMS applied over the visual cortex on sensorimotor control in individuals with recurrent lateral ankle sprains.

## Data Availability

The data that support the findings of this study are available from the corresponding author upon reasonable request.
